# Unveiling with Density Functional Theory the Optical Property Variations of Three Kinds of Graphene for Acetaminophen Sensor Design

**DOI:** 10.1021/acsomega.4c06168

**Published:** 2024-12-26

**Authors:** Ali Fransuani Jiménez González, Daniel E. Ceballos Herrera, Roberto G. Ramírez-Chavarría, Rosa M. Ramírez Zamora, Luis Fernando Magaña Solís

**Affiliations:** † Instituto de Ingeniería, Universidad Nacional Autónoma de México, código postal 04510, Mexico City 01000, Mexico; ‡ Instituto de Física, Universidad Nacional Autónoma de México, código postal 04510, Mexico City 01000, Mexico

## Abstract

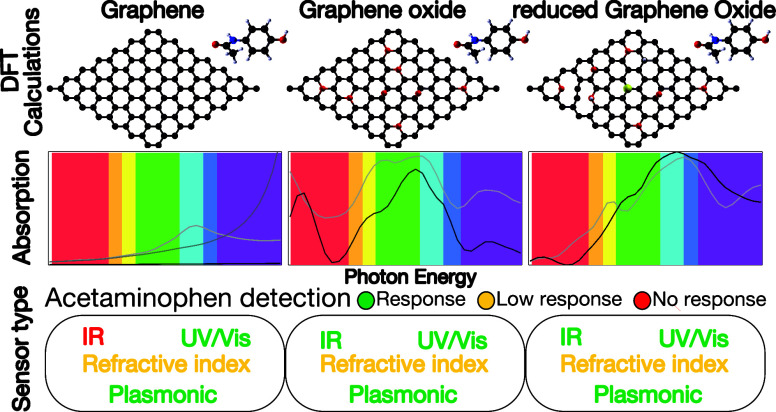

Understanding the interactions between molecules and sensing elements is crucial to improving sensors. We present one step toward getting closer to the breach between theory and empirical sensor development. Through density functional theory (DFT) calculations, we explored the changes in some optical properties of pristine graphene (G), graphene oxide (GO), and reduced graphene oxide (rGO) interacting with one molecule of acetaminophen (APAP). The main goal is to unveil which graphene—G, GO, and rGO—works better as a substrate to detect APAP for sensor applications based on UV/vis and near-infrared absorption, refractive index changes, and plasmon resonances. For this effort, we used Quantum ESPRESSO software. We calculated each supercell’s adsorption energy and recovery time containing the APAP and one graphene variant. Afterward, we calculated the optical absorption, refractive index, reflectivity, and electron energy loss spectroscopy (EELS), a technique to identify the material’s plasmons. Then, we analyzed the changeovers in the optical properties mentioned for each graphene layer with and without APAP. We found that G works for UV/vis and plasmon sensors operating in blue and UV–C regions; GO has the highest performance range in UV/vis and plasmonic sensors operating in UV–C; rGO has the highest performance range in UV/vis sensors working on near-infrared and UV–C. Additionally, rGO plasmonic sensors present an “oscillation of percentage variations” from visible to UV. Our study offers a strategy for creating a map to select the best substrate to develop selective APAP optical sensors.

## Introduction

Acetaminophen (APAP) is one of the four active pharmaceutical ingredients (APIs) with concentrations of about micrograms across all continents.^1^ Furthermore, APAP toxicity is the second most common cause of liver transplantation worldwide and the most common cause of liver transplantation in the USA.^2^ Thus, early detection and good sewage treatment are necessary for people’s health, so we chose this API for detection.^3,4^ On the other hand, experimental costs for sensor development can be very high. Likewise, the lack of synergy between theory and experiment limits the strategic development of more efficient sensors. Molecular simulations are an alternative to guide the experimental part and technological development, such as those proposed in this research, due to their low relative cost and excellent approximation to experimental results. Many researchers based their calculations only on the absorption energy, band structure, or density of states. Here, we want to contribute with research that uses more detailed ab initio calculations.^5^ These computational tools have much more power that should be used to create intelligent strategies for sensor development.

Additionally, this simulation can provide clarity and more detail regarding what happens in optical sensors, allowing more effective proposals to complement technological development. One promising material for sensor development is graphene^6−9^ because of its unique properties.^10−12^ We aim to unveil which type of graphene —pristine graphene (G), graphene oxide (GO), and reduced graphene oxide (rGO)— works better in detecting APAP in four sensors: UV/vis, refractive index, plasmonic, and near-infrared. We used density functional theory (DFT) with Quantum ESPRESSO software^13−15^ to understand better the optical variations of each graphene interacting with APAP.

## Results and Discussion

### Optimized Structures

**Figure 1:**
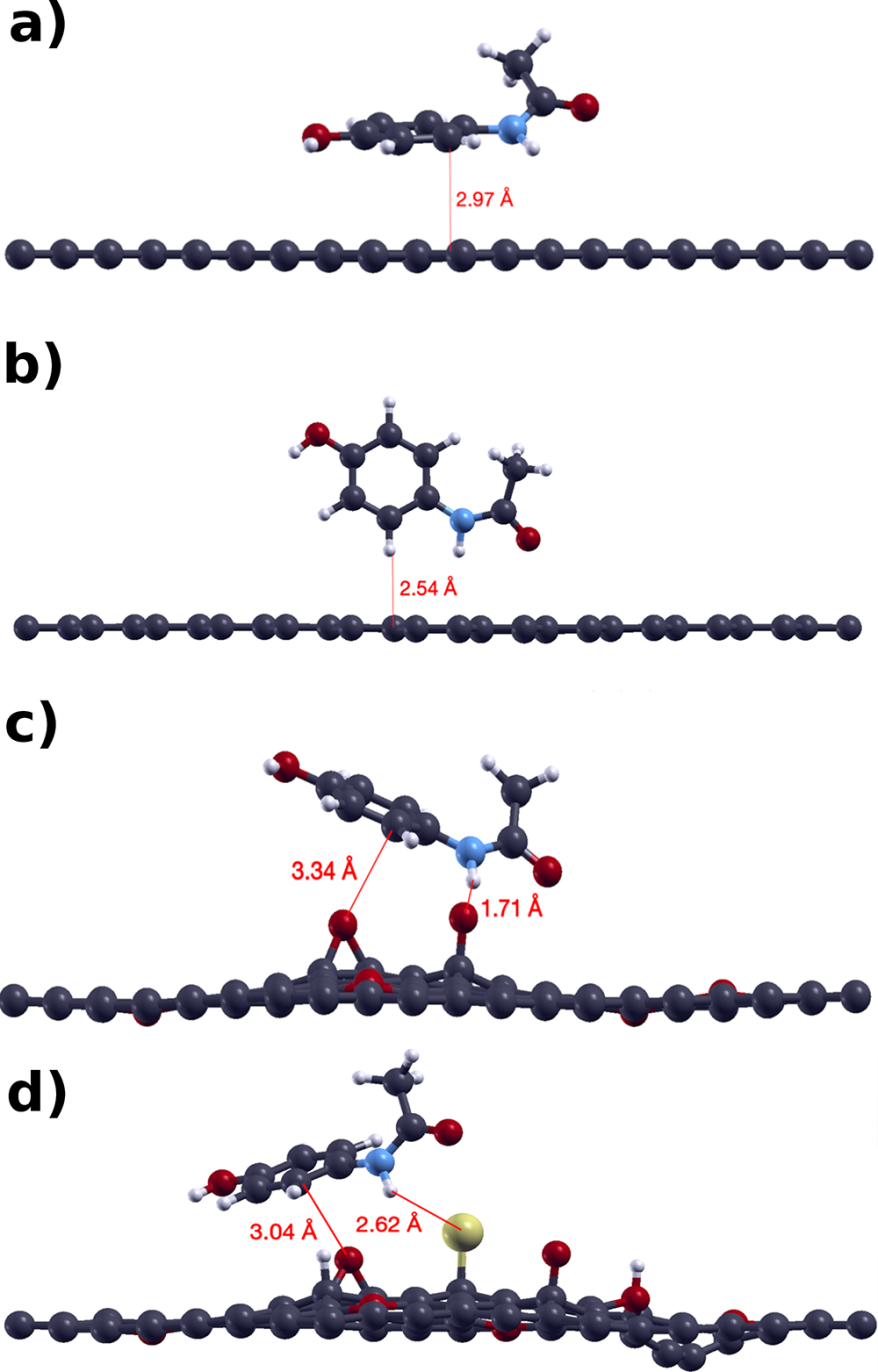
Final coordinates of each geometrical optimization: (a) G interacting with APAP horizontally, (b) G interacting with APAP vertically, (c) GO interacting with APAP horizontally, and (d) rGO interacting with APAP horizontally. The red line denotes some distances between acetaminophen and graphene. We erased the supercell for visualization purposes.

We performed a geometrical optimization for each sheet of pristine graphene (G), graphene oxide (GO)—with 8% oxygen—and reduced graphene oxide (rGO). For rGO, we set the closest atom percentages from the Shilpent technical datasheet,^16^ standing our percentages used: 90.3% carbon, 6.8% oxygen, 0.97% sulfur, and 1.9 hydrogen. Shilpent’s analysis indicates ∼0.4% N; our previous work^17^ found that nitrogen percentages less than 1% do not affect the graphene’s optical properties, so we discarded this atom in this research. Additionally, we optimized only one acetaminophen molecule (APAP) to avoid self-interactions. This is an essential step in finding the minimum energy geometry of each substrate and each acetaminophen individually. The Fermi energies obtained in eV were APAP = −4.249, G = −2.961, GO = −2.367, and rGO = −2.949. Afterward, we built a supercell with each graphene layer and one APAP and set it to 3 Å from the nearest atom from G, GO, or rGO. Figure S1 illustrates the initial configuration for all of the structures. Then, we performed a second geometrical optimization, setting all angles and axes to move but maintaining the lattice invariant—removing all system tensions. Additionally, this second calculation allows us to calculate the Fermi energy for each interacting system, which will be used to shift the band structure and density of states and quickly identify the conduction and valence bands. The Fermi energies (in eV) found in the interacting systems are G+APAP = −3.3144, GO+APAP = −2.2867, and rGO+APAP = −2.6624. Details of the approximations used can be found in Section 1 of the Supporting Information. Figure 1 depicts the optimized structures for the three cases and the APAP approach to the sheets.

For G, we try two APAP orientations: with the carbon ring horizontal and vertical to the graphene’s surface; both final configurations can be found in Figure 1. As we mentioned, APAP’s initial distance is 3 Å from the closest graphene atom. Thus, the APAP horizontal final distance from graphene is 2.97 Å (Figure 1 a), which means APAP gets closer to 0.03 Å to the pristine graphene. Meanwhile, vertical APAP gets closer to 0.46 Å of graphene’s layer (Figure 1b). In Figure 1c, APAP gets closer to 1.29 Å to the GO’s nearest atom, one oxygen, and Figure 1d shows APAP getting closer to S about 0.38 Å. As shown below, these distances are enough to present a van der Waals interaction, confirmed with adsorption energies. We will discuss it when talking about the projected density of states. We calculated the adsorption energies using the equation:
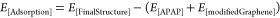
1

Our results (see Table 1) indicate that APAP horizontal to G is more stable, so we used only this configuration for GO and rGO structures. This choice was made to save computational resources. Considering the adsorption energies in Table 1, we calculated the recovery time using the equation (eq S1) from Section 1 of the Supporting Information.

**Table 1:** Values of the Adsorption Energies and Recovery Time (τ) for Each Layer Interacting with Acetaminophen

structure	*E*_Abs_ (eV)	τ (s)	τ (s) at *T* = 100 °C
G+APAP	–0.55	2.5 × 10^–5^	3.2 × 10^–6^
G+APAP_vertical_	–0.35	1.0 × 10^–7^	6.0 × 10^–9^
GO+APAP	–1.51	3.5 × 10^12^	3.1 × 10^7^
rGO+APAP	–1.19	1.3 × 10^7^	1.4 × 10^3^

An optimal recovery time should be between 1 × 10^–2^ and 1 × 10^2^ s for good detection. If we consider this suggested range, then pristine graphene cannot hold the APAP enough time to be detected; this indicates that a cavity or an additional treatment, such as atom doping, should be done on the graphene substrate to improve the recovery time. GO and rGO present an ample recovery time, which means that the APAP will be highly adsorbed to the surface, which could saturate the sensor.

We also made a theoretical supposition, calculating the recovery time to 100 °C to analyze the possibility of reusing the GO and rGO substrates in sensors. As shown in Table 1, it is possible to heat the rGO to 100 °C, and the APAP molecule will unbind the surface in a lapse of 23 min. On the other hand, if we set *T* = 200 °C, the APAP will unbind from the GO surface in a lapse of 1.29 × 103 s, which means 21.5 min.

### Bands Structure

Then, we calculated the band structure depicted in Figure S2 of the Supporting Information. For pristine graphene, upon interaction with APAP, two semiflat bands appear at 0.96 and 1.78 eV; these correspond to a break in the system’s symmetry caused by the presence of APAP. The properties and characteristics of the pristine graphene bands remain intact. Bands are highly dependent on the system’s symmetry. In the case of rGO, they show practically pure separated bands due to the misshaping presented in rGO (Figure 1d), where, in addition to presenting a bulk in the central area, the in-plane oxygen causes an opening in the graphene sheet. It is interesting to observe a gap in the bands; considering the κ and Γ points of greater symmetrical interest, we have openings of 0.224 and 0.126 eV, respectively. On the other hand, GO with oxygen at 8% presents a band opening of 0.236 and 0.391 eV on the κ and Γ points, respectively. Those gaps will cause relevant performance in the optical properties. For a better understanding of the interactions between layers and APAP, we performed subsequent calculations.

### PDOS

**Figure 2:**
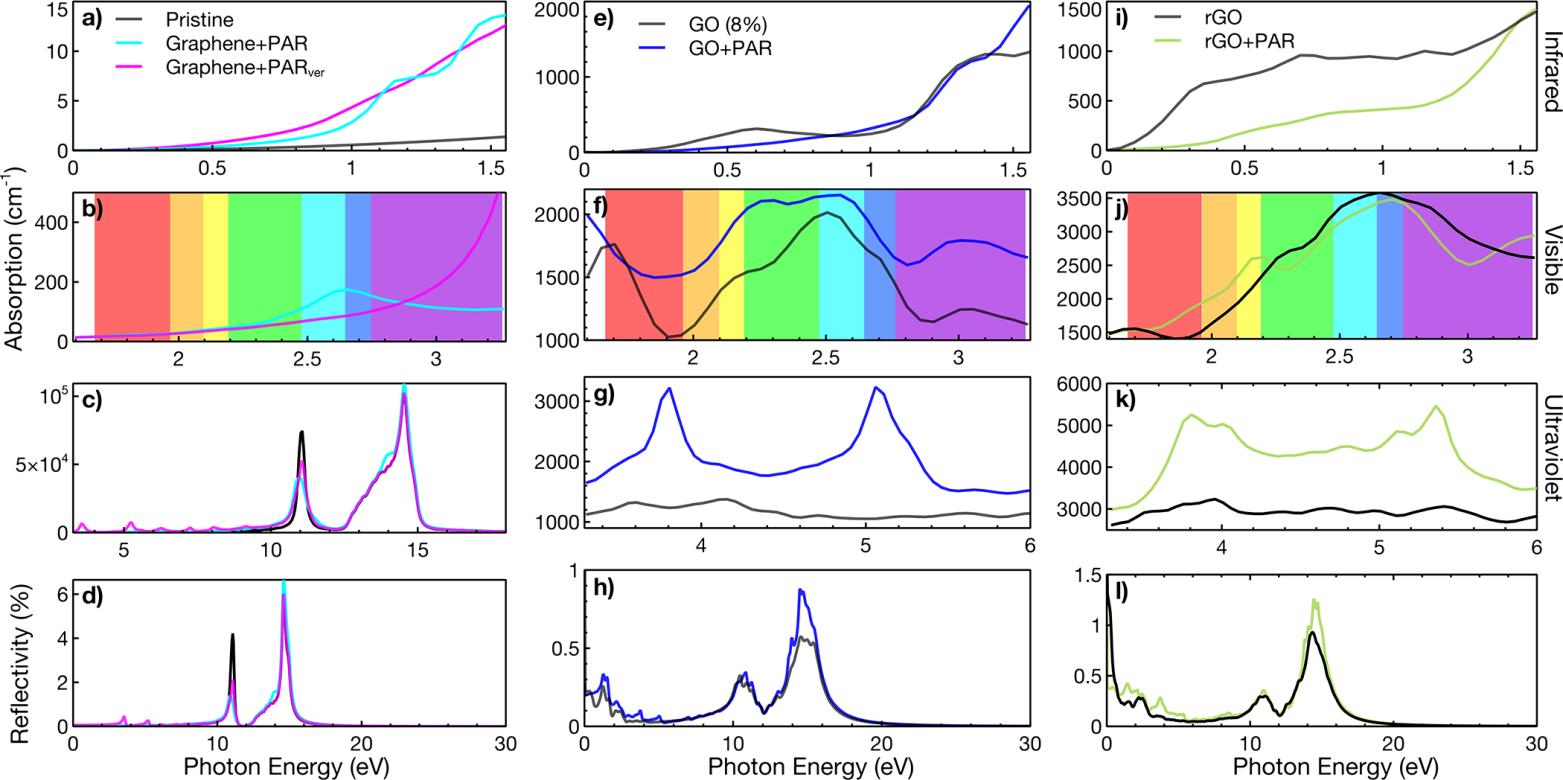
IR absorption for (a) G with APAP horizontally and vertically, (e) GO, and (i) rGO. The visible absorption for (b) G with APAP considering two orientations, (f) GO and (j) rGO. The UV absorption for (c) G with APAP considering two orientations, (g) GO and (k) rGO. The reflectivity for (d) G with APAP considering two orientations, (h) GO and (l) rGO.

We obtained the projected density of states (PDOS) for all of the calculated structures shown in Section 4 of the Supporting Information. In Figure S3, the APAP oxygen is the closest to the G layer—its distance is 3.2 Å. It corresponds to the oxygen next to the carbon ring (nonacetyl). Together with the nitrogen, their orbital p shifts to more profound energy from −2.26 to −2.69 eV, and only the nitrogen p orbital shifts from −1.41 to −1.74 eV. This shift means that G transfers the charge to APAP, confirming the van der Waals interaction mentioned. It is well-known that the dipole-selection rules for isolated graphene layers vanish in the frequency region below 10 eV. At these energies, all of the transitions correspond to π bands. Therefore, the appearance of the two peaks at −0.93 and −1.74 eV in the PDOS shown in Figure S3 is responsible for the increased optical absorption in the visible and IR regions. It should be noted that the p orbitals of the benzene ring, interacting with the π electrons of graphene, facilitate such an optical variation. For GO’s PDOS, the oxygen plotted (Figure S4) corresponds to the APAP’s acetyl group and strongly interacts with the one GO oxygen. They interchange charge; the acetyl oxygen transfers the charge to the GO. The shift to the highest energy for the acetyl oxygen is depicted in Figure S4, going from −2.53 to −1.17 eV. When GO interacts with APAP, we also observe in Figure S4 that the ring is still responsible for creating new states near the Fermi level, thus broadening the optical absorption in the visible region—as we will see later in Figure 2. On the other hand, Figure S4 shows the electronic exchange between the O of acetyl and the O of GO at an energy of −1.17 eV. These new states are likely responsible for the absorption peak at 3.8 eV, corresponding to UV-A. Again, for the rGO (Figure S5), we analyzed the oxygen from APAP’s acetyl group. This oxygen is the closest to the S atom of the rGO; its distance is 3.1 A. We only considered the p orbital since it has the strongest interaction. We noticed that the oxygen in the acetyl group of APAP transfers charge to sulfur from reduced graphene oxide (rGO). This transfer is shown by a shift of its p orbital to lower energies from −0.36 to −1.58 eV and the hybridization at energies of −1.39 and −1.58 eV. This electronic adjustment resulted in the formation of four states near the Fermi level, whereas only one state was observed at the Fermi level in rGO alone. This redistribution allows for lower energy transitions and is reflected in the IR absorption, as shown in Figure 2. Lastly, in all cases, the interaction between paracetamol and the electronic exchange of the benzene ring leads to increased optical absorption in the UV, corresponding to peaks near 5.1 eV. At the same time, N interacts with the three p orbitals through an H-bridge. The carbon ring of APAP contributes practically nothing to the interaction with rGO; the sum of its p states remains at the same energy.

On the other hand, the oxygen of the rGO to which APAP comes closest participates to a lesser extent in the van der Waals interaction; this is observed when moving to a deeper energy, from −2.43 to −2.75 eV. The van der Waals interactions and shifts of states allow significant and detectable changes in the optical properties shown below.

### Dielectric Tensor

We plotted the dielectric function of pristine graphene (Figure S6) from 0 to 20 eV to compare and validate our calculations. The dielectric tensor for small in-plane momentum transfer has the same behavior exhibited in Marinopoulos’ work.^18^ Subsequently, we apply eqs 2–5 shown in Section 1 of the Supporting Information, obtaining each supercell’s refractive index (RI), optical absorption, reflectivity, and electron energy loss spectroscopy (EELS). For the optical absorption and reflectivity, we only consider the propagation of the electromagnetic wave perpendicular to the graphene plane, and we drill down to optical absorption into three regions: infrared (IR), visible (Vis), and ultraviolet (UV); see Figure 2.

Marinopoulos’ work^18^ only gives the dielectric function for in-plane light propagation; hence, the light propagation parallel to the *c*-axis (perpendicular to the graphene surfaces) is directly compared with the UV absorption spectrum shown in Figure 2c, where we can observe the same peaks at 11 and 14 eV. The reflectivity for the three cases are plotted from 0 to 30 eV, showing that all layers remain almost transparent for all electromagnetic spectra analyzed. Finally, we analyzed the light propagation in the three directions for RI and EELS and only plotted the regions of interest from 0.7 to 6 eV—meaning from near-infrared to far UV. We present all graphics for RI and EELS in the Supporting Information; here, we only display tables summarizing the essential percentage changes.

### Optical Absorption and Reflectivity

This section discusses the variations in optical absorption between each graphene without and with APAP. We present only the absorption perpendicular to the surface, which means the *z*-axis. We plotted near-infrared spectroscopy from 0.496 to 1.589 eV. The visible region is considered from 1.65 to 3.26 eV. The ultraviolet region is plotted from 3.26 to 18 eV. The reflectivity is plotted from 0 to 30 eV, and it is shown only for the complement. We also show that G interacts with APAP horizontally and vertically. Still, our discussion will focus only on the horizontal orientation due to its highest energy stability. Also, for G with APAP, we omitted the analysis of the changes in IR because the variations are less than 35 cm^–1^.

We found that APAP increases the absorption in the visible region of G by more than 1000%, reaching the central peak in the blue region shown in Figure 2b. Meanwhile, the ultraviolet and reflectivity vary by less than 10%. It is worth mentioning that APAP vertically presents a much better perpendicular light absorption to the plane from 3 eV (violet) up to 5.26 eV. It reaches its maximum at 3.55 eV (UV–C) with a difference of 3.6 × 10^3^ % compared with the absorption of APAP oriented horizontally. Moreover, APAP oriented vertically presents a difference of 7.8 × 10^4^ % from pristine G. However, as recovery time suggests, this signal could be intermittent due to the short time of adherence to the surface. Generally speaking, Figure 2f shows that GO with APAP presents more changes in the visible region, with the lowest change of 9% and the maximum of 48.7%. Also, we can observe that GO interacting with APAP presents an inversion of light absorption in the IR for APAP detection with a minimum percentage change of 9.5% and a maximum of 90.8%. Furthermore, GO and rGO present a striking peak reduction of about 0.43 eV due to the N–H of APAP and 0.42 eV because of the O–H bond. Finally, Table 2 summarizes the main differences between the absorption spectra of G, GO, and rGO interacting with APAP in all regions depicted in Figure 2. We want to highlight that rGO presents a good absorption at the red laser energy of 1.9 eV.

**Table 2:**
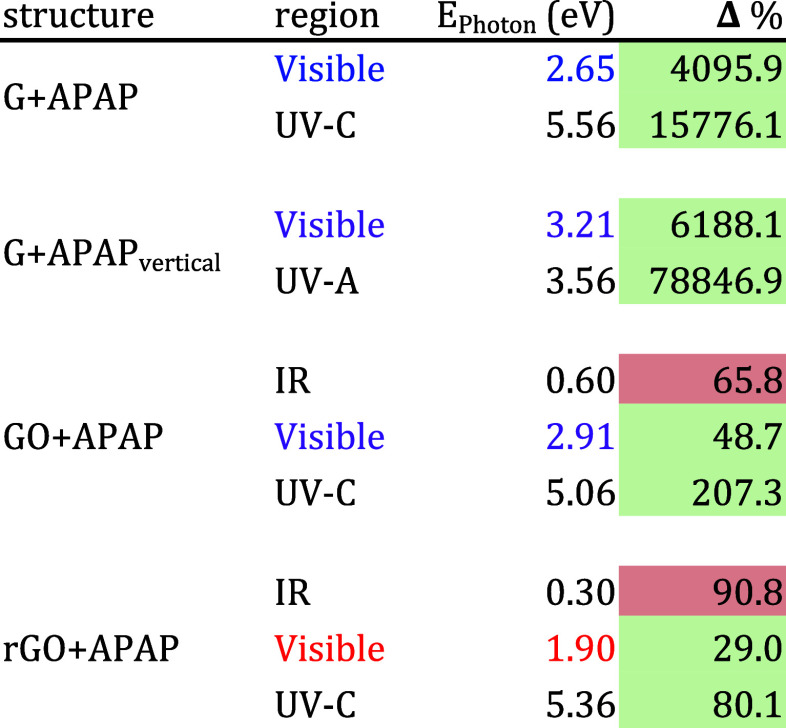
Representative Values of Percentage Changes in Optical Absorption for Each Layer Interacting with APAP^a^

a A green cell indicates a positive percentage increment. Meanwhile, a red cell indicates a percentage reduction in light absorption. We suggest the color absorption region with the color letter.

### Refractive Index

It is known that graphene has complex refractive indices in some regions of the visible and UV.^19,20^ However, due to the methodology used in calculating the refractive index (eq 4, Supporting Information), some areas are less than 1. Due to the limitations of our calculations, an approximation that allows the calculation of complex refractive indices is necessary. We can rely on the results obtained since the dielectric function Figure S6 for “small in-plane” coincides with the results of the calculations performed by Marinopoulos.^18^Figures S7–S9 show the refractive index from near-infrared to far UV (0.7 to 6 eV) for each graphene layer with and without APAP. Here, we present a summary in Table 3 with the main percentage variations depicted in the figures mentioned. We will discuss only the regions with refractive index higher than 1.

**Table 3:**
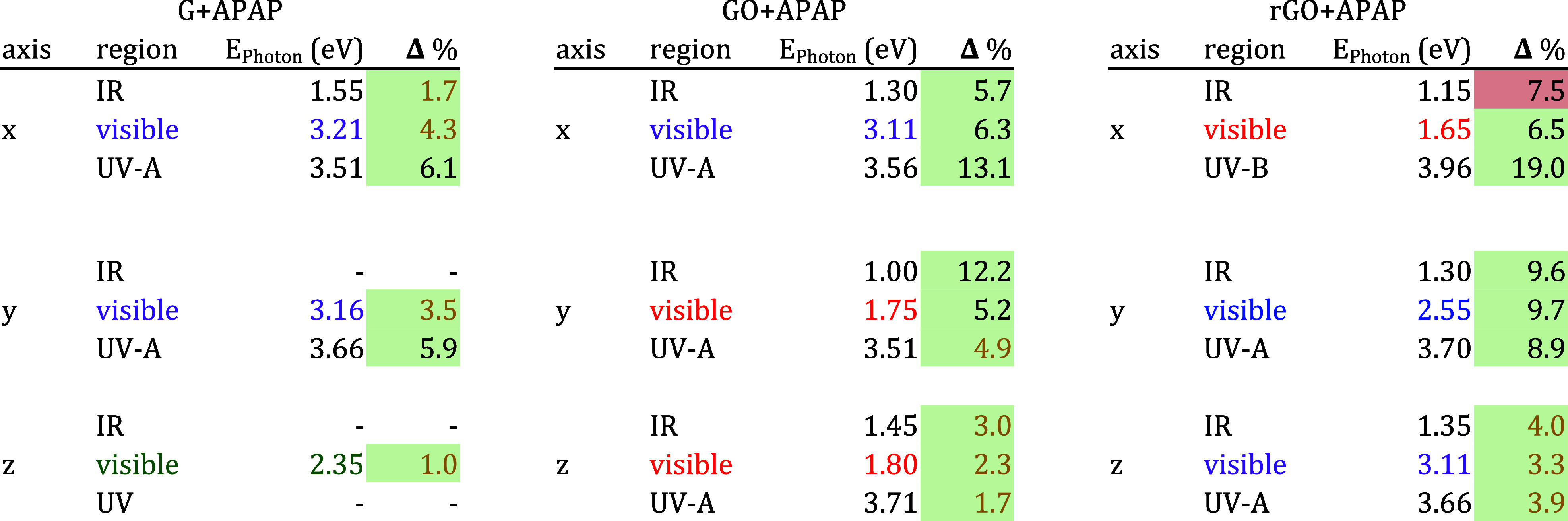
Representative Percentage Changes in Refractive Index for Graphene’s Layers Interacting with APAP^a^

a A green cell indicates a positive percentage increment. Meanwhile, a red cell indicates a percentage reduction in the refractive index. We only considered a refractive index greater than 1. We suggest the color absorption region with the color letter. Finally, we use yellow letters for percentage variations between 1% and 5%.

These variations are detectable but should only be taken as potential detection; they need more accurate approximations. The calculations in this work are handy in suggesting ranges for sensors to detect higher refractive index changes. Also, the percentages above 10% are only for electromagnetic waves propagating in the plane, and most sensors work with perpendicular propagation.

### EELS

This section discusses electron energy loss spectroscopy (EELS). This technique measures the energy loss of electrons as they interact with the sample to identify the material’s plasmons. Figures S10–S12 show the EELS from near-infrared to far UV (0.7 to 6 eV) for each graphene layer with and without APAP. Here, we present a summary in Table 4 with the main percentage variations depicted in the figures mentioned. The analysis suggests that APAP can induce plasmon resonance variations of around 10% in the visible and UV regions for the three graphene sheets analyzed; these changes occur in all three directions. For G interacting with APAP with in-plane propagation, these percentage variations of over 10% occur only in UV regions. For the IR and visible regions, the percentage changes are less than 10%.

**Table 4:**
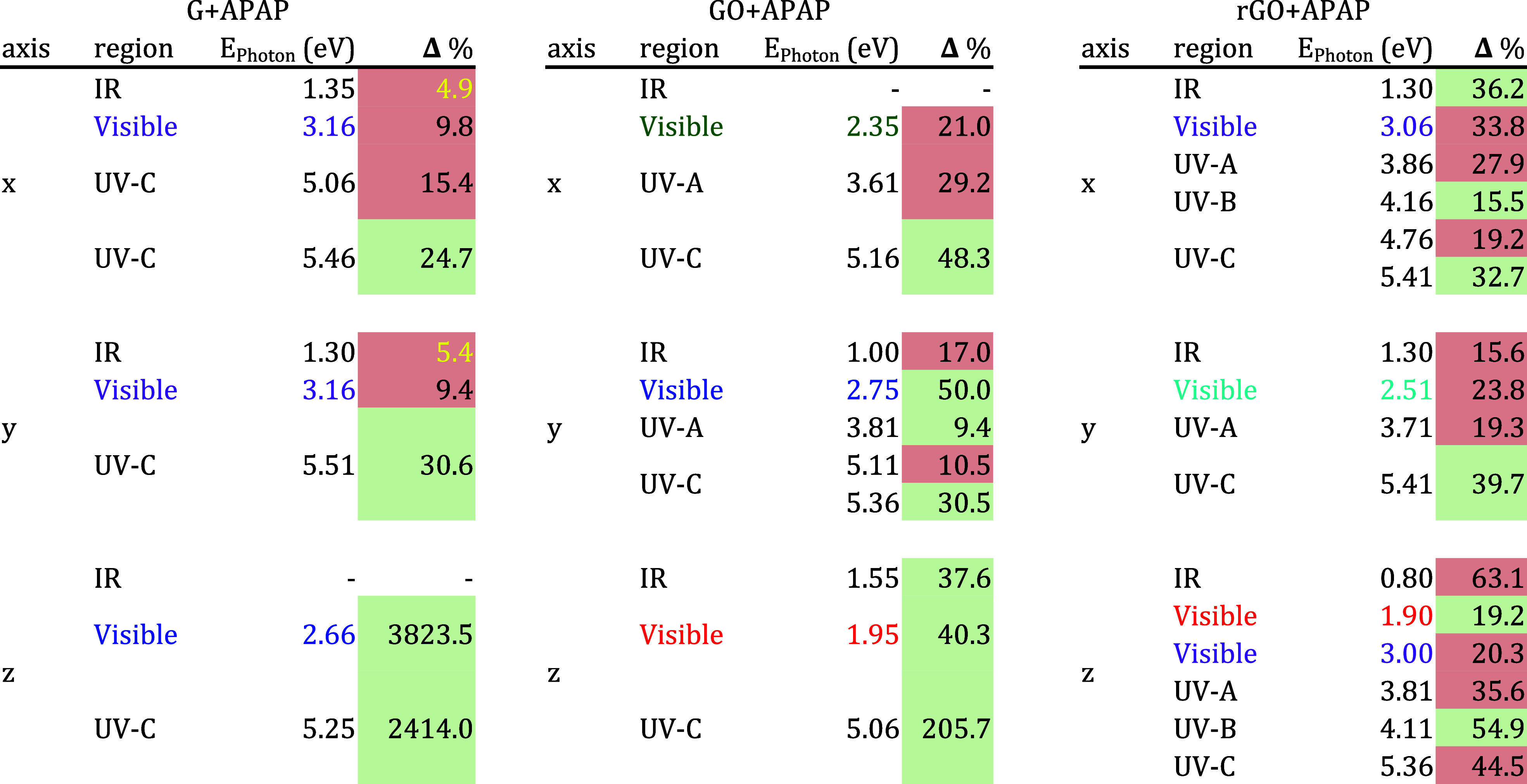
Representative Percentage Changes in Electron Energy Loss Spectroscopy (EELS) for Each Graphene’s Layers Interacting with APAP^a^

a A green cell indicates a positive percentage increment. Meanwhile, a red cell indicates a percentage reduction in plasmon resonance. We suggest the color absorption region with the color letter. Finally, we use yellow letters for the percentage variations between 1% and 5%.

In Figure S10, the plasmon activity from the red region can be appreciated with a percentage change of 1000% for a photon energy of 1.8 eV. This percentage change will increase to the maximum peak shown in Table 4.

Meanwhile, the maximum peaks reached for APAP vertically are with photon energies of 3.26, 3.56, and 5.36 eV, with changes over 6.7 × 10^4^ %. On the other hand, APAP interacting with GO and rGO produces plasmon resonance changes from yellow to green regions (2.0–2.35 eV) with changes of around 22%. Moreover, the presence of APAP in GO and rGO produces periodic variations in the EELS at small energy intervals (see how black and blue curves alternate periodically in Figures S11 and S12; also see the red and green alternate in Table 4). It impacts the formation of plasmons with periodic variations of the intensity in the photon energy range of Vis and UV. This spectral behavior can represent a kind of fingerprint characteristic of APAP—which can help develop selective APAP sensors.

In summary, Table 5 shows the value in which each graphene sheet has the highest percentage change for each kind of sensor analyzed detecting APAP. This table only considers data from an incidence normal to the graphene plane since it is the predominant detection incidence. In addition, for GO and rGO, the values shown in this summary for the IR region differ from those in Table 1 because we consider the standard operating ranges of the infrared sensors. Furthermore, both IR values for GO correspond to the openings in Figure 2e with percentage variations over 28%. On the other hand, IR values of rGO are the photon energy limits showing a percentage change over 15% (see Figure 2i). For the plasmon column, we indicate the photon energies for the highest peaks in Figures S10–S12.

**Table 5:**
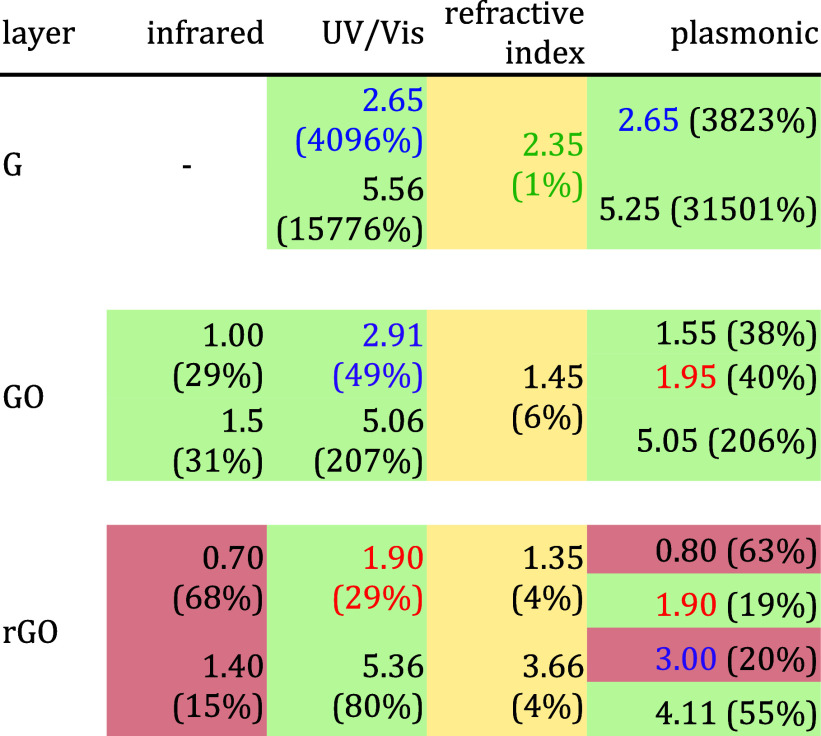
Summary of Photon Energies (eV) with the Best Performance for Each Kind of Sensor Detecting APAP^a^

a In parentheses, the percentage change is indicated. We only considered normal light incidence. A green cell indicates a positive percentage increment. Meanwhile, a red cell indicates a percentage reduction. We suggest the color absorption region with the color letter and use yellow cells for percentage variations between 1 and 5%.

## Conclusions

Recovery time suggests graphene oxide (GO) and reduced graphene oxide (rGO) sensor saturation during APAP detection. Meanwhile, pristine graphene needs a cavity or doping.

We found that pristine graphene (G) works for UV/vis and plasmon sensors operating in blue and UV–C regions with a percentage change of over 4000%. Also, G responds better to vertical APAP. However, it is less stable, so its signal may be intermittent.

GO and rGO can work in three of the four selected sensors: infrared, plasmonic, and UV/vis. GO has the highest performance range in UV/vis and plasmonic sensors operating in UV–C, with a percentage change of over 200%. Likewise, both kinds of sensors can work in visible regions with a percentage change of 40%.

rGO has the highest performance range in UV/vis sensors working on near-infrared (0.70 eV with a percentage change of 68%) and UV–C (5.36 eV with a percentage change of 80%). Additionally, plasmonic sensors present an “oscillation of percentage variations” going from visible to UV with percentage changes of over 20%.

Reduced graphene oxide (rGO) is more adaptable when considering a broader photon energy range for acetaminophen detection. Furthermore, we can analyze the range and intensity of the responses. rGO exhibits an excellent range of responses to energy photons, while graphene oxide (GO) shows a more significant percentage variance. Therefore, the choice of the best substrate will depend on the user’s preferences, based on whether it will work at a simultaneous multifrequency or prefer a higher percentage variation.

This research is valuable because it is one formal development approach to guide the conscious design of sensors. It also offers a strategy for creating a map to select the best substrate while developing a specific sensor. Much more work and calculations are needed, even to clarify the effects of symmetry and the percentages of dopants.

## Supporting Information Available

Computational details, additional DFT calculation, and complementary graphics (PDF)
