# Supplementary material for: Unveiling with Density Functional Theory the Optical Property Variations of Three Kinds of Graphene for Acetaminophen Sensor Design
Source: ACS Omega. 2024 Dec 26;10(1):318–24. doi: 10.1021/acsomega.4c06168 (PMC11739940; doi:10.1021/acsomega.4c06168)
Supplement: Supplementary file 1 — ao4c06168_si_001.pdf [file ao4c06168_si_001.pdf]

Supporting Information

Unveiling with DFT the optical properties variations of three kinds of graphene for acetaminophen-sensor design

Ali Fransuani Jiménez González[a]*, Daniel Enrique Ceballos Herrera[a], Roberto G. Ramírez-Chavarría[a] , Rosa María Ramírez-Zamora[a], and Luis Fernando Magaña Solís[b]

[a] Instituto de Ingeniería, Universidad Nacional Autónoma de México, Apartado Postal 20-364, Mexico City 01000, Mexico.

[b]Instituto de Física, Universidad Nacional Autónoma de México, Apartado Postal 20-364, Mexico City 01000, Mexico

*Correspondence author's mailing address: Instituto de Ingeniería, Universidad Nacional Autónoma de México, código postal 04510, Mexico City 01000, México.

Email: AJimenezG@iingen.unam.mx

1. DFT Calculations

For visualizations, we used the XCrySDen software.[1] For all calculations, we used Quantum Espresso v.7.0 (QE) software,[2–4] which works with the density functional theory (DFT) under the approximation of plane waves and pseudopotential formalism. We performed all calculations under the generalized gradient approximation (GGA) with the Perdew-Burke-Ernzerhof (PBE) expression for the exchange-correlation functional.[5] We also considered norm-conserving pseudopotentials[6] (necessary for optical calculations) with the Trouiller-Martins method[7] and the Grimme correction[8–11] for the Van der Waals interactions. We choose 8$\times$8$\times$2 k-point mesh within the Monkhorst-Pack scheme.[12] We set an energy cut-off value of 80 R for plane waves and the energy convergence threshold for self-consistency to 10^{-8} Ry.

As the QE considers periodic boundary conditions, the supercell size is large enough to avoid spurious interactions (see Figure S1). Similarly, we chose the cell parameter on the z-axis to 15 Å; in this way, we can model a 2D surface in a vacuum.

We optimized each graphene surface with the selected parameters for the three cases: pristine graphene, graphene oxide, and reduced graphene oxide. Then, we included the APAP at 3 Å and performed a geometric optimization to minimize the system's forces below a threshold of 1.0 $\times 10^{-3}$ Ry/Bohr.

At the end of each optimization, we get the adsorption energy, and utilizing Eyring's theory of state transitions,[13] we calculated the recovery times to estimate the absorption time of the APAP on each graphene surface. The equation used is:

$$\tau = \left(\frac{h}{K_b T}\right) e^{\frac{-E_{ADS}}{K_b T}} \text{ ...(Eq S1)}$$

Here, E_{ads} is the adsorption energy of each graphene layer interacting with one acetaminophen; h and K_B are Plank and Boltzmann's constants, respectively. Finally, T is the temperature. We must mention that we do not perform molecular dynamics calculations in this work. The T = 300 K value was only used in equation (6) to estimate the recovery time at room temperature.

Afterward, we performed self-consistent and non-self-consistent (with 18╳18╳1 k-point mesh) calculations on the resultant structures to obtain their corresponding band structures, the optical properties, the density of states (DOS), and the projected density of states (PDOS). From the band structure, we calculated the imaginary part of the dielectric tensor ($\Im$m $\varepsilon_{ii}(\omega)$). Then, we used the Kramers-Kroning relation to calculate the real part of the dielectric tensor ($\Re$e $\varepsilon_{ii}(\omega)$). Having the two parts of the dielectric tensor, we calculated the reflectivity ($R_{ii}(\omega)$) and optical absorption ($A_{ii}(\omega)$) with the following equations:[14]

$$R_{ii}(\omega) = \frac{(n-1)^2+K^2}{(n+1)^2+K^2} \text{ ... (Eq S2)} \qquad A_{ii}(\omega) = \frac{2\omega K(\omega)}{c} \text{ ... (Eq S3)}$$

Where n_{ii} is the refractive index and k_{ii} is the extinction coefficient, given by:

$$n_{ii}(\omega) = \frac{\sqrt{|\varepsilon_{ii}(\omega)|+\Re\varepsilon_{ii}(\omega)}}{2} \text{ ...(Eq S4)} \qquad k_{ii}(\omega) = \frac{\sqrt{|\varepsilon_{ii}(\omega)|-\Re\varepsilon_{ii}(\omega)}}{2} \text{ ...(Eq S5)}$$

2. Optimized Structures

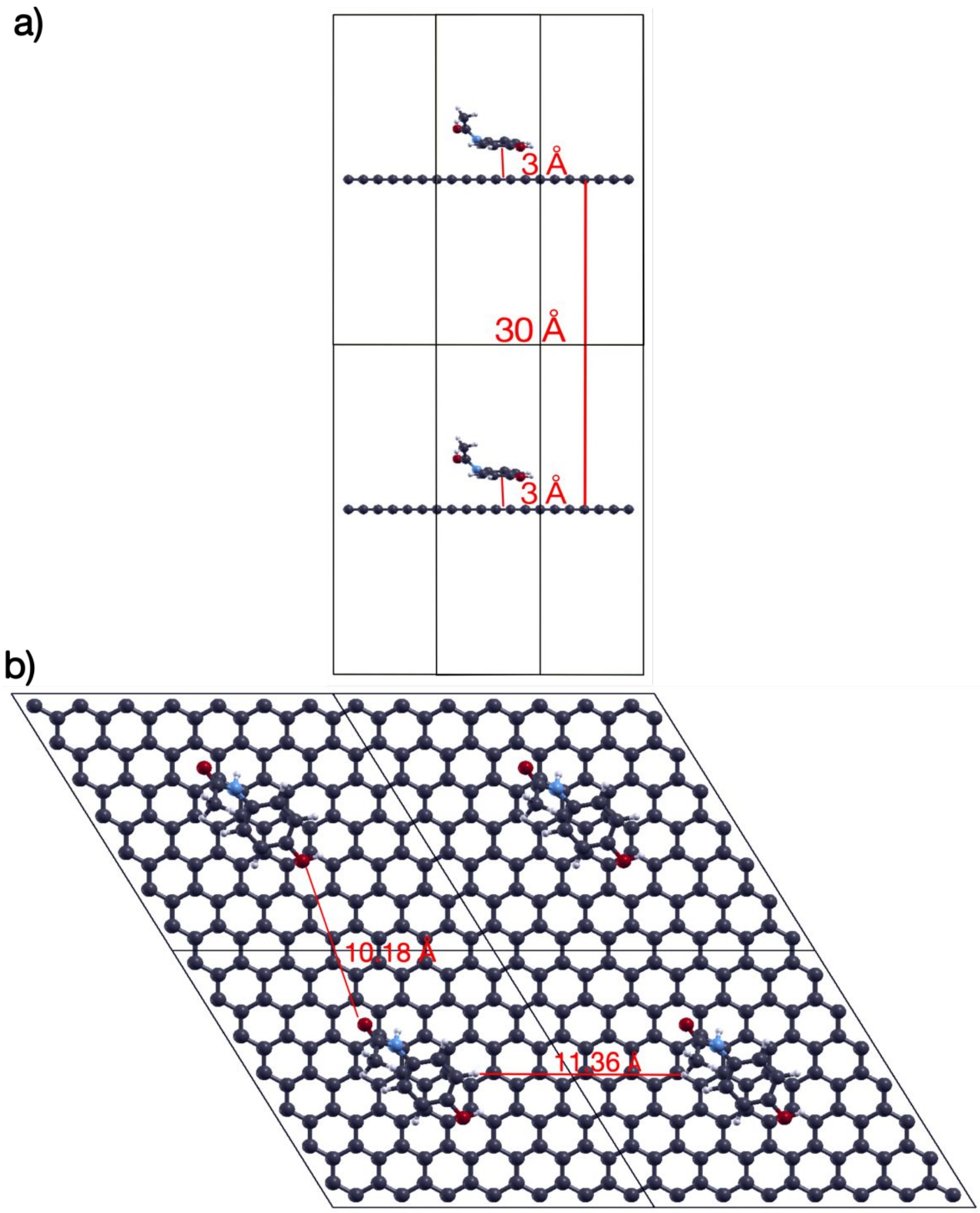

Figure S1. Initial configurations for the structural optimizations performed with pristine graphene interacting with acetaminophen. We set all graphene configurations in a similar distribution. a) shows the distance of 30 Å between each acetaminophen and the upper graphene sheet. b) shows a top view with a 2×2 supercell repetition. We put a distance of over 10 Å between acetaminophen to avoid spurious interactions.

3. Band Structure

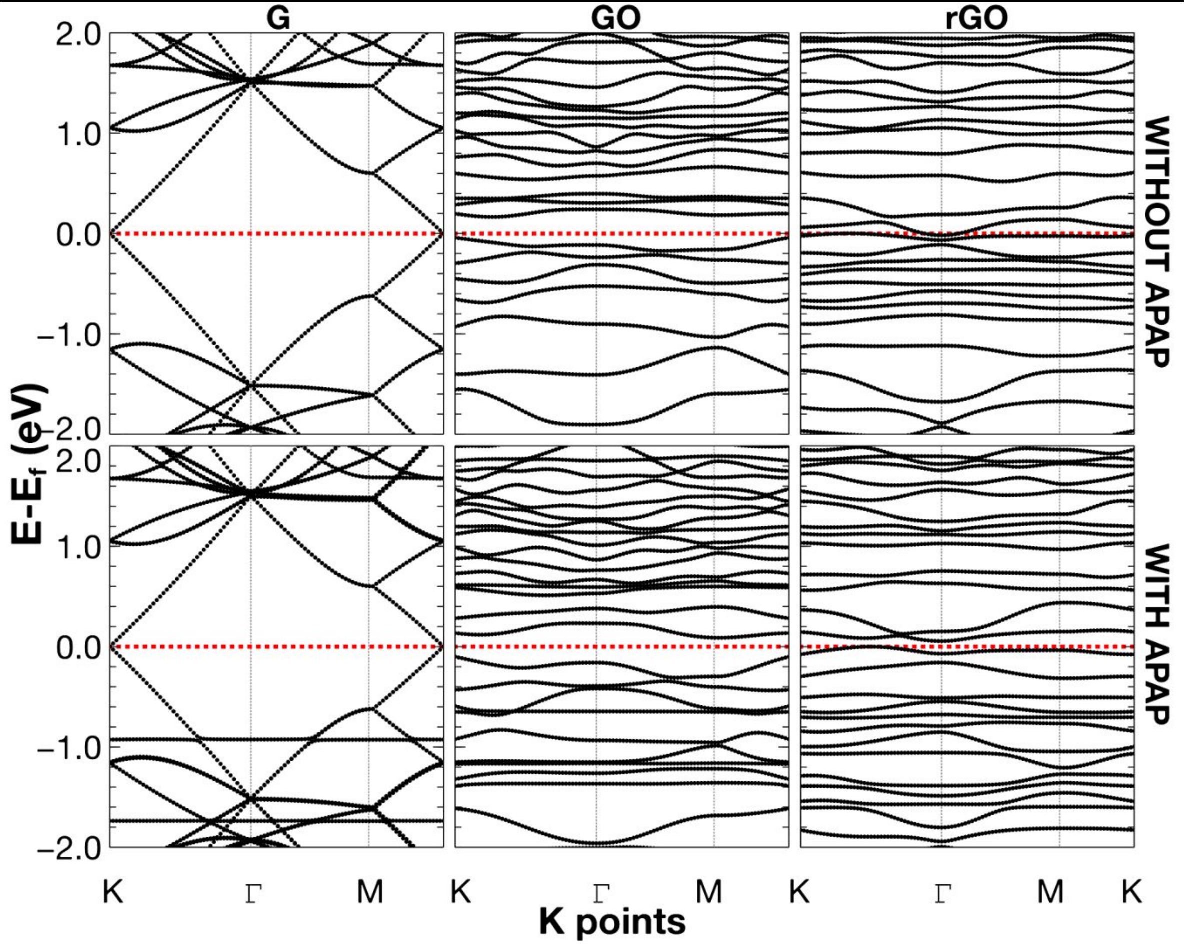

Figure S2. At the top, we show the band structures of pristine graphene (G), graphene oxide (GO), and reduced graphene oxide (rGO). At the bottom, we show the band structures of each layer interacting with acetaminophen (APAP).

4. PDOS

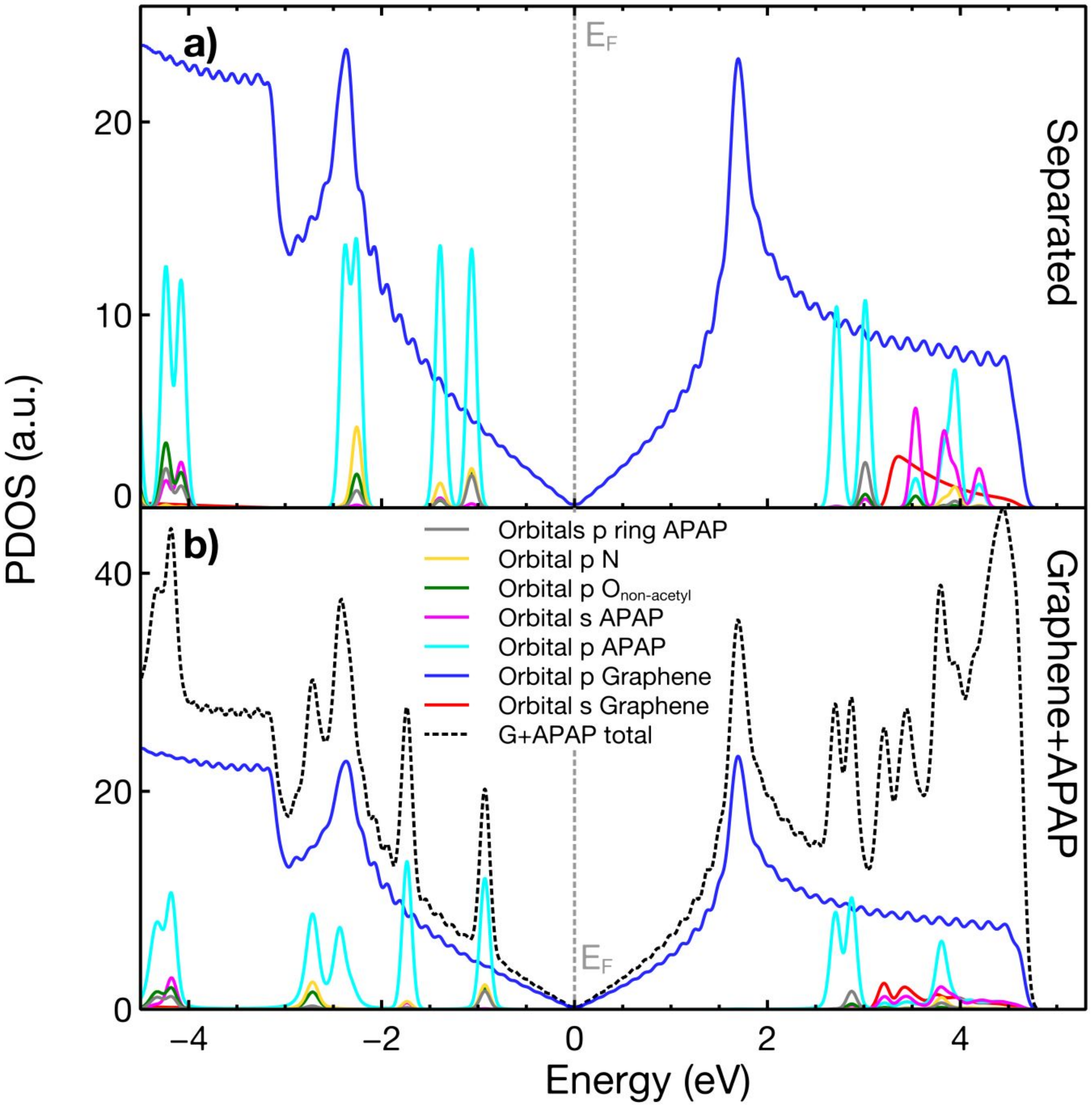

Figure S3. a) Projected density of states (PDOS) for pristine graphene and acetaminophen (APAP) on different unit cells. b) PDOS for pristine graphene interacting with one APAP. The plot shows the variations on each s and p orbital for the cases considered.

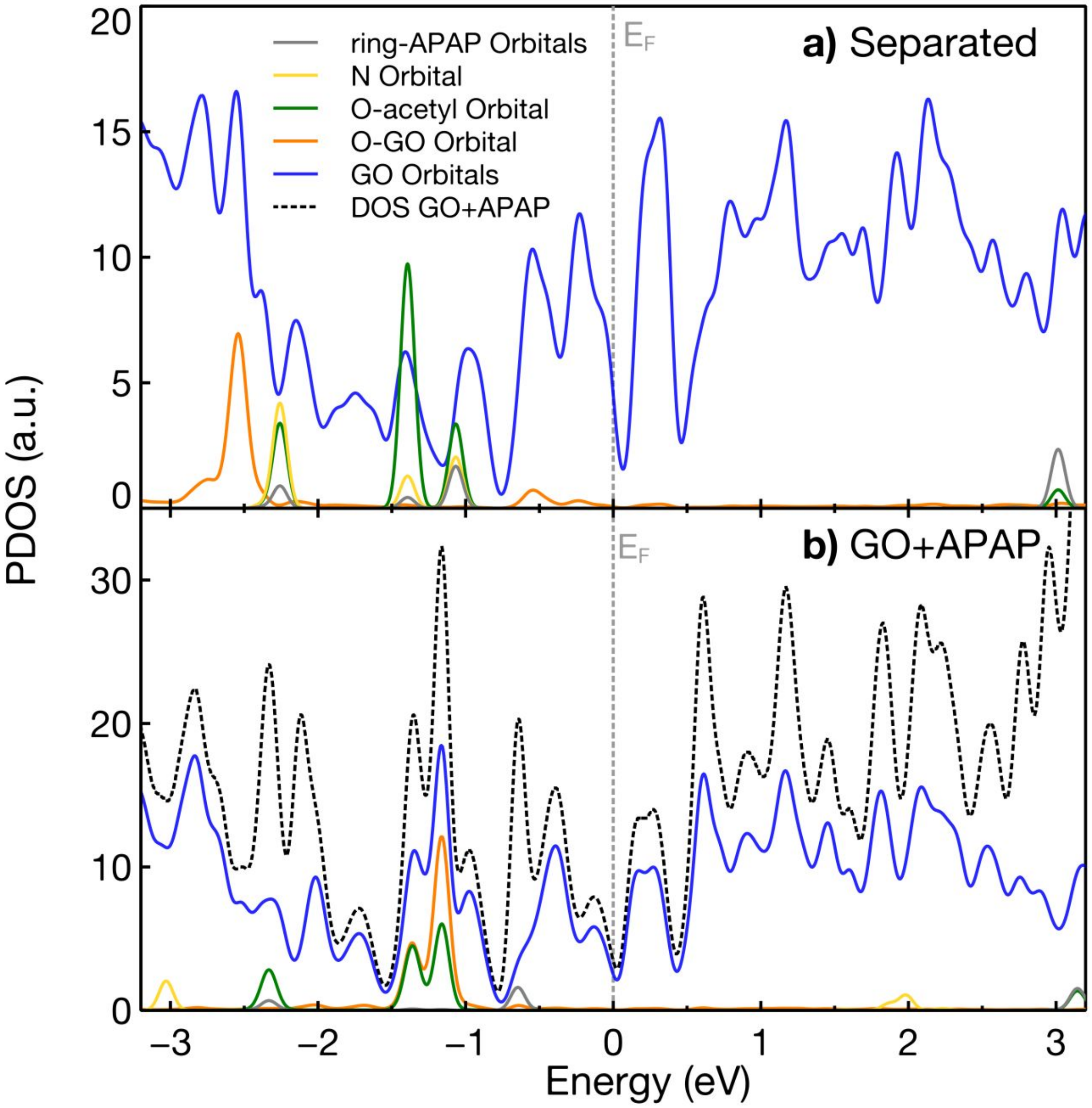

Figure S4. a) Projected density of states (PDOS) for graphene oxide (GO) and acetaminophen (APAP) on different unit cells. b) PDOS for GO interacting with one APAP. The plot shows the variations only for the p orbital for each atom plotted. We removed the total APAP PDOS and GO s-orbital for image clarity.

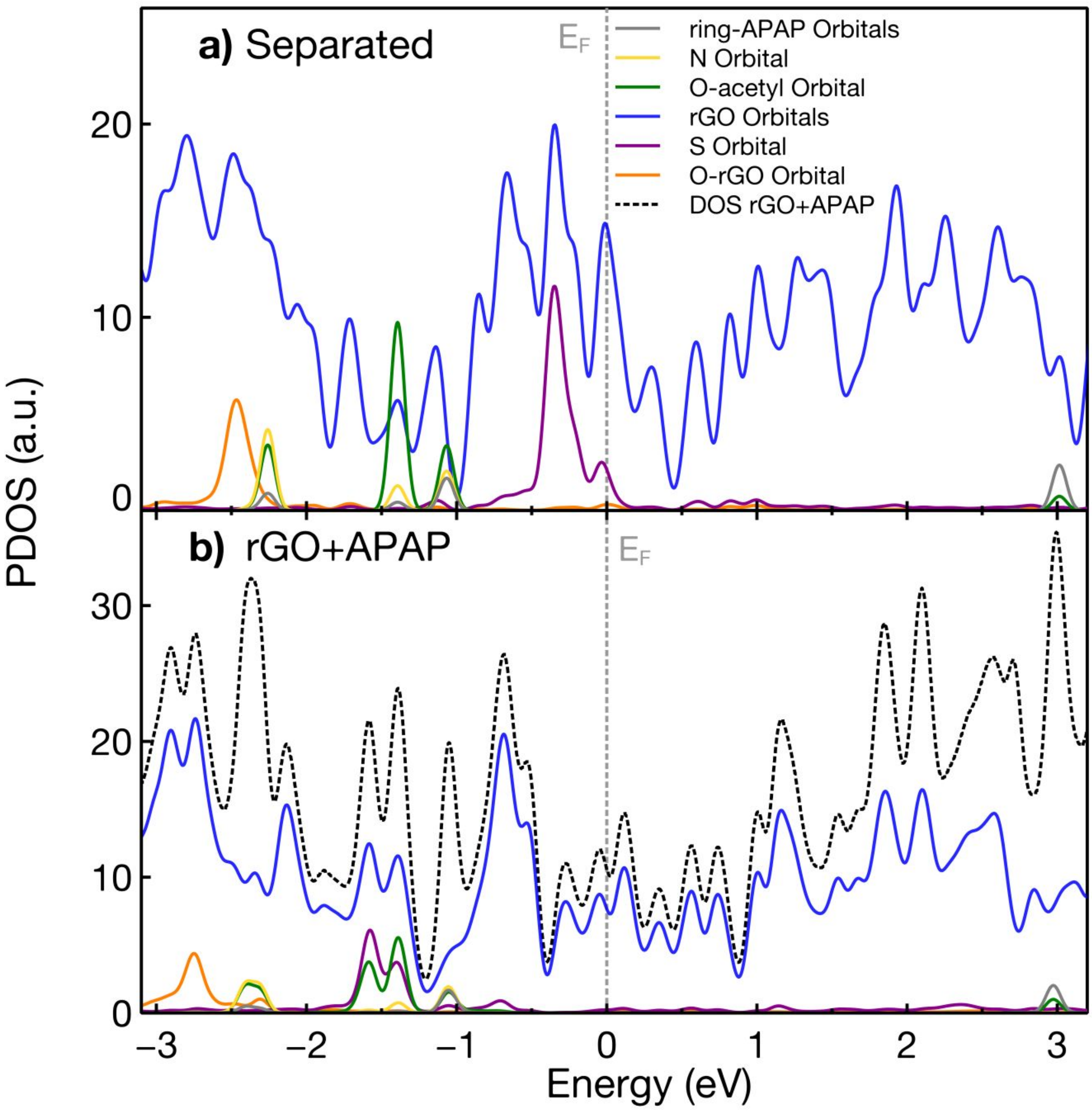

Figure S5. a) Projected density of states (PDOS) for reduced graphene oxide (rGO) and acetaminophen (APAP) on different unit cells. b) PDOS for rGO interacting with one APAP. The plot shows the variations only for the p orbital for each atom plotted. We removed the total APAP PDOS and rGO s-orbital for image clarity.

5. Dielectric tensor

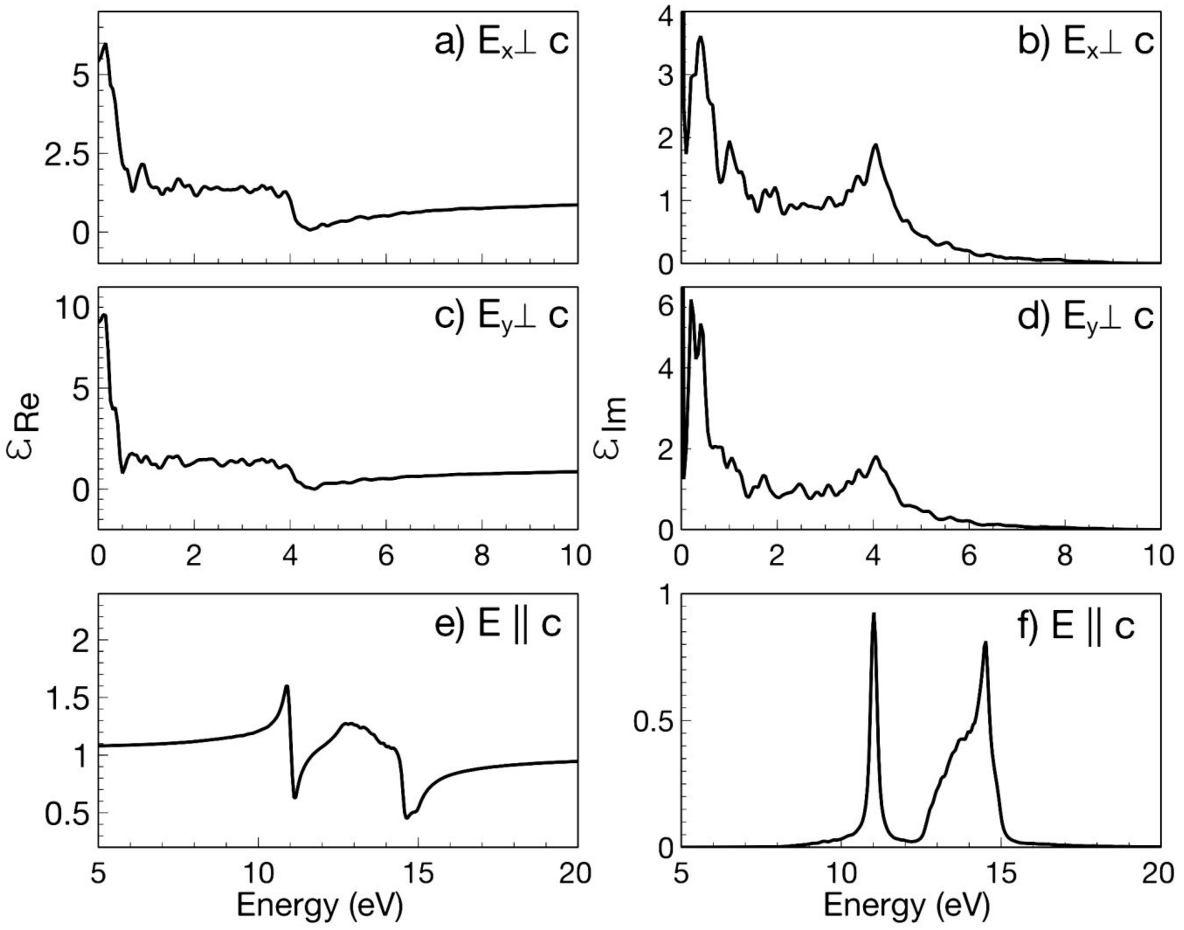

Figure S6. The dielectric function for pristine graphene. The left column shows the real part of the dielectric tensor, while the right column depicts the imaginary part. a), b), c), and d) are the in-plane propagation of light. e) and f) are the light propagation perpendicular to the surface. The notation used is for easy comparison with Marinopoulos' work.[15] It refers to the electric field vector perpendicular or parallel to the c-axis (same as the z-axis).

5.1 Refractive index

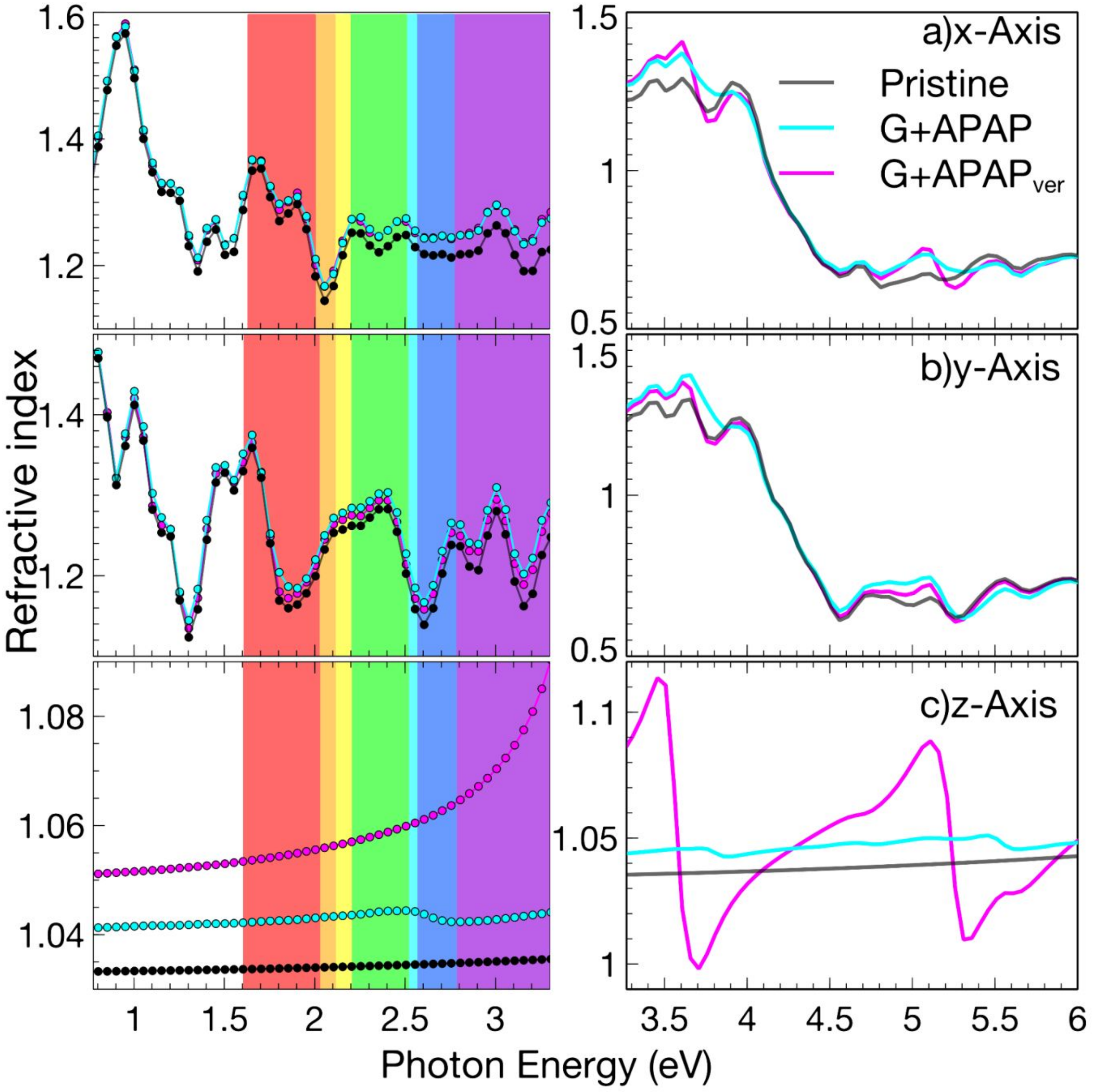

Figure S7. The refractive index for pristine graphene without APAP and with APAP horizontally and vertically to the plane. Each row shows one of the three axes.

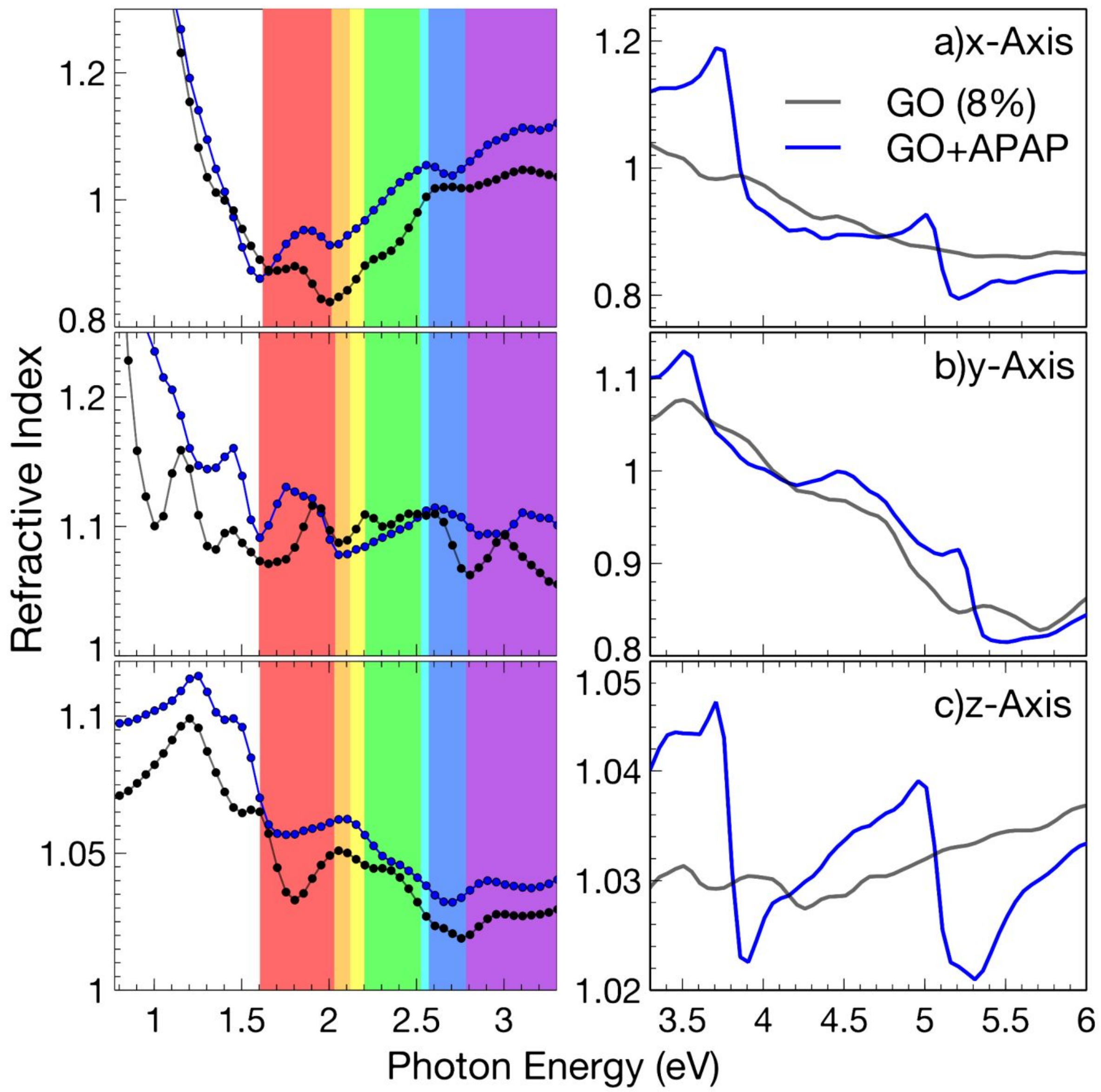

Figure S8. The refractive index for graphene oxide without APAP and with APAP. Each row shows one of the three axes.

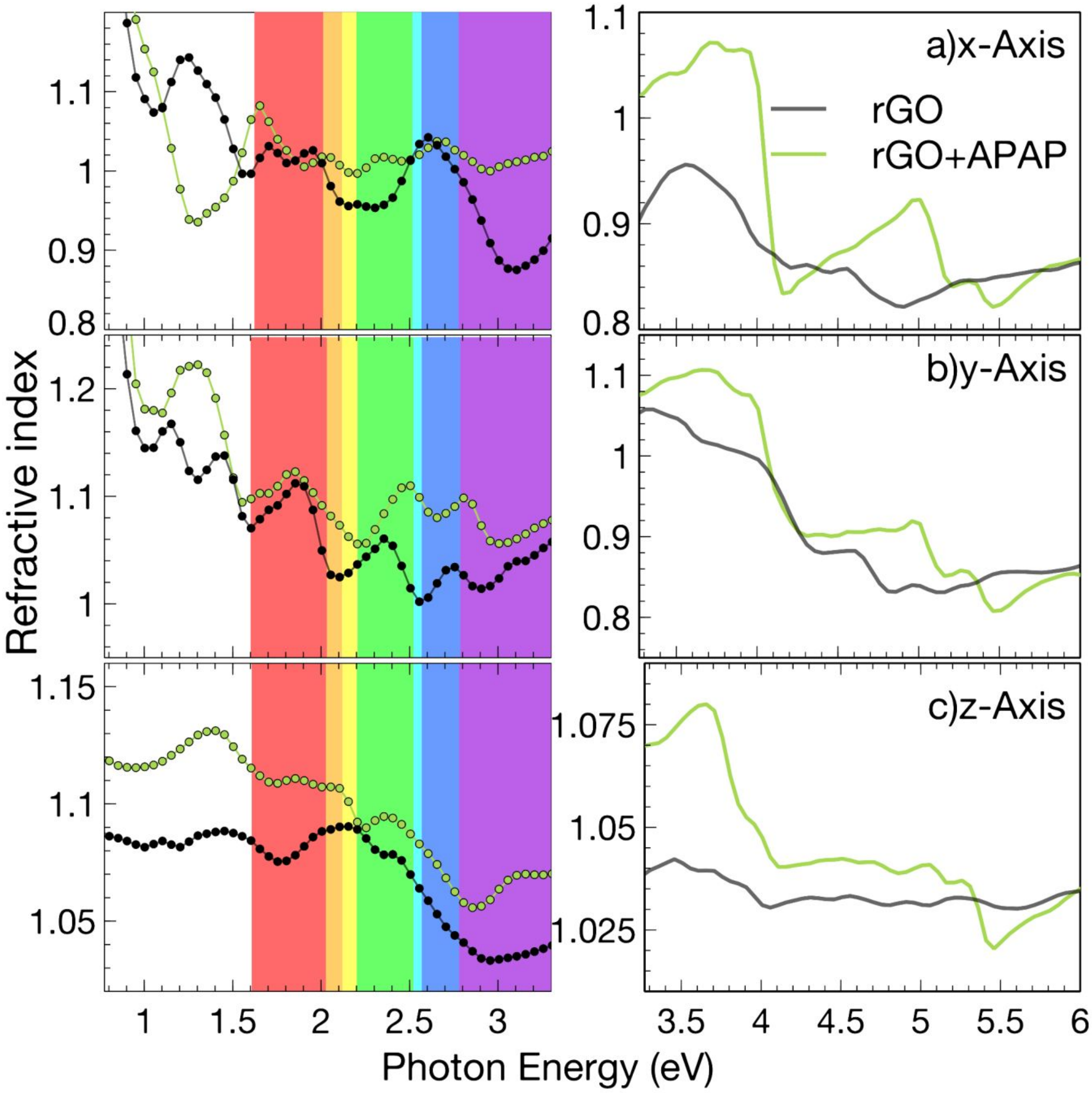

Figure S9. The refractive index for reduced graphene oxide without APAP and with APAP. Each row shows one of the three axes.

5.2 Electron Energy Loss Spectroscopy

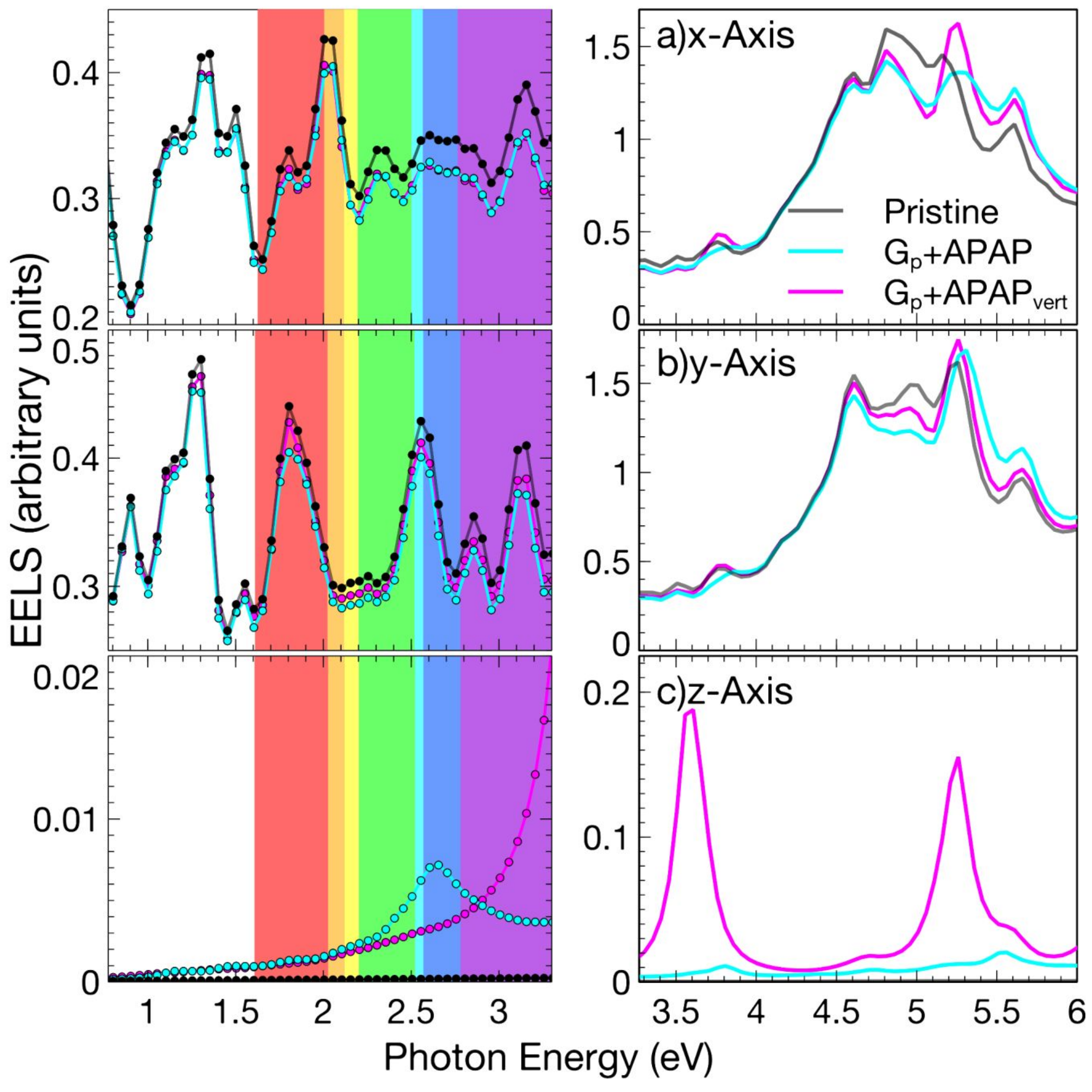

Figure S10. Electron Energy Loss Spectroscopy (EELS) of pristine graphene without APAP and with APAP horizontally and vertically to the plane. Each row shows one of the three axes.

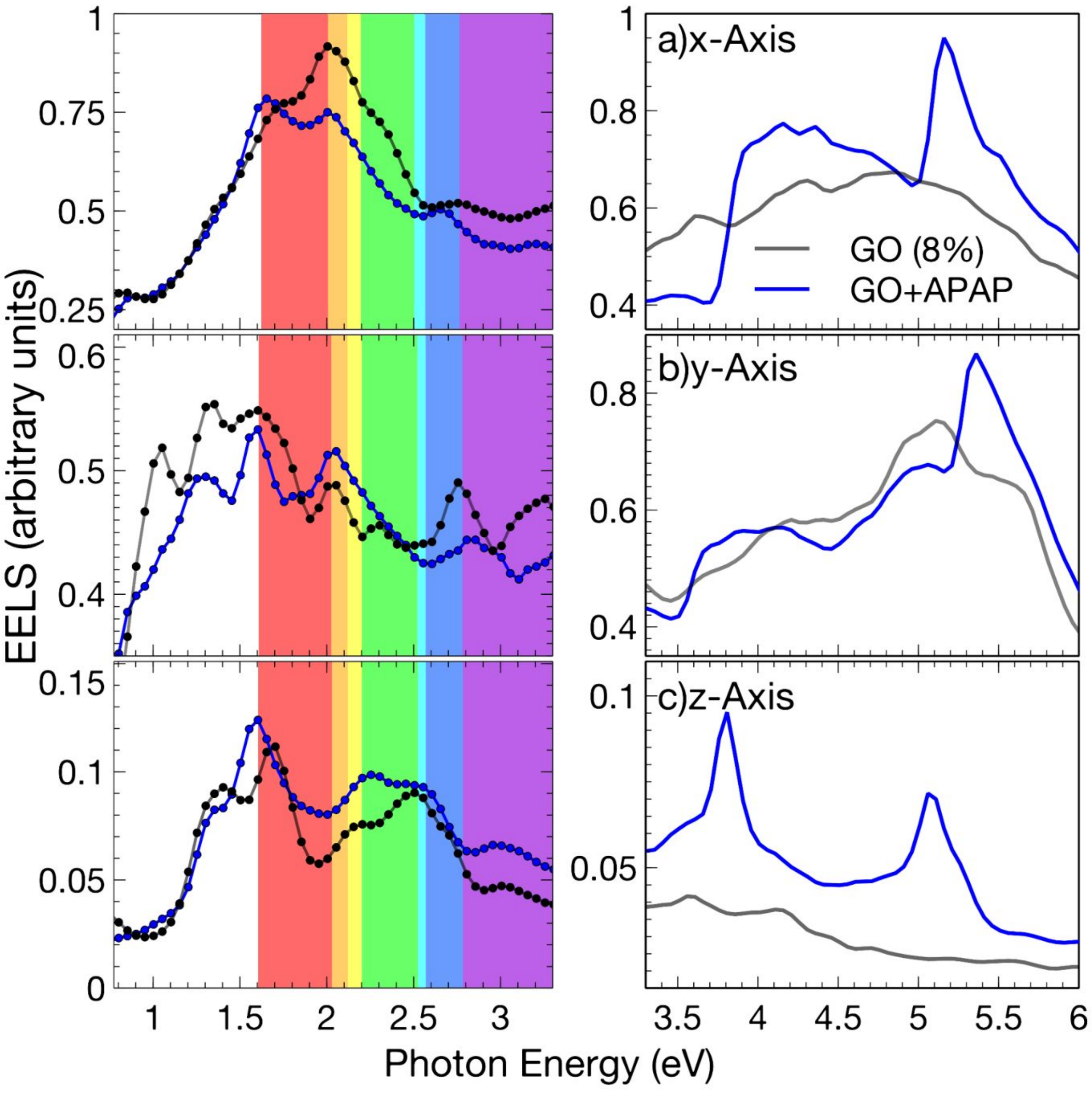

Figure S11. Electron Energy Loss Spectroscopy (EELS) of graphene oxide without APAP and with APAP. Each row shows one of the three axes.

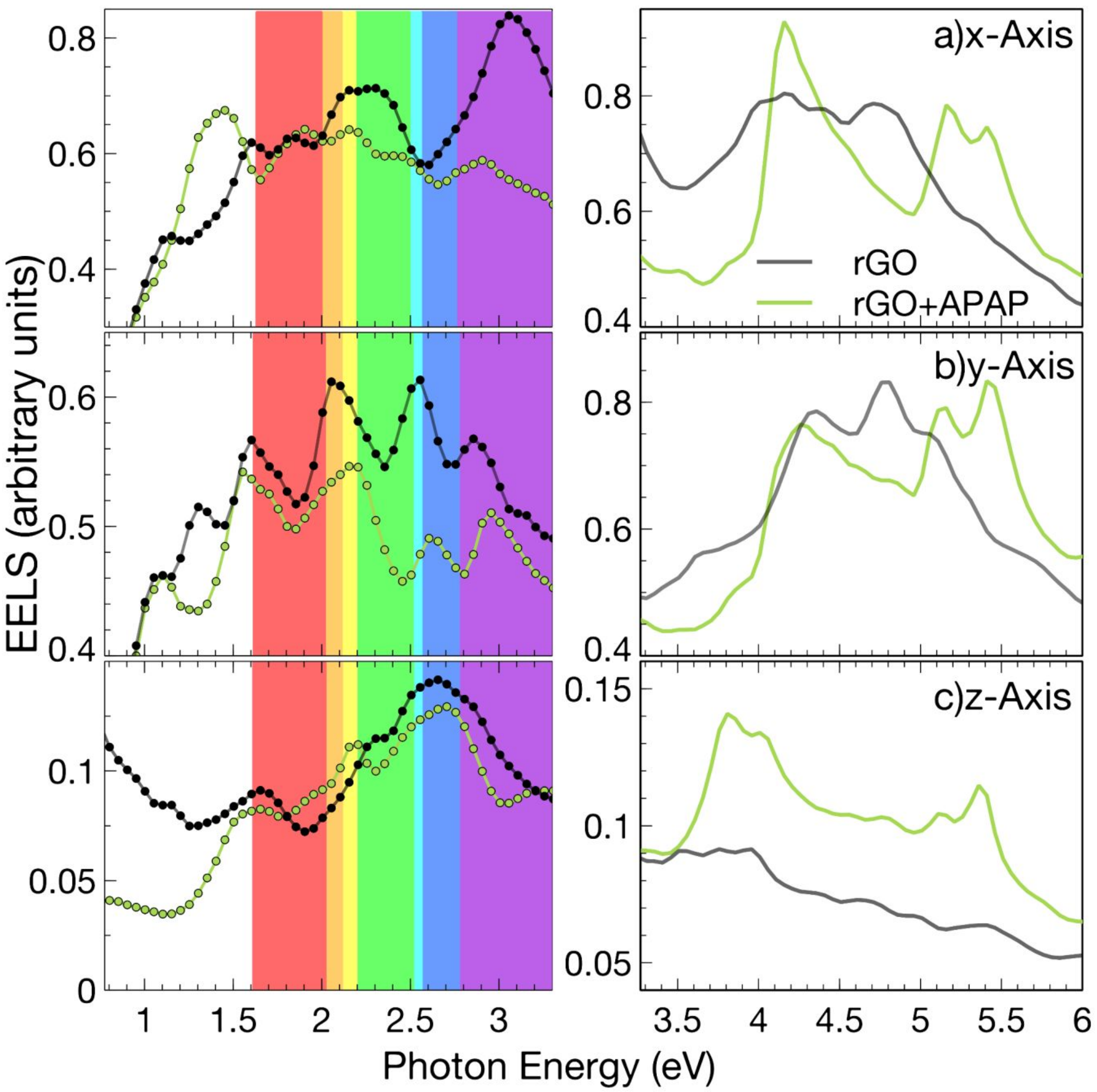

Figure S12. Electron Energy Loss Spectroscopy (EELS) of reduced graphene oxide without APAP and with APAP. Each row shows one of the three axes.

REFERENCES

(1) Kokalj, A. XCrySDen—a New Program for Displaying Crystalline Structures and Electron Densities. *J. Mol. Graph. Model.* **1999**, *17* (3), 176–179. https://doi.org/10.1016/S1093-3263(99)00028-5.

(2) Giannozzi, P.; Baroni, S.; Bonini, N.; Calandra, M.; Car, R.; Cavazzoni, C.; Ceresoli, D.; Chiarotti, G. L.; Cococcioni, M.; Dabo, I.; Dal Corso, A.; De Gironcoli, S.; Fabris, S.; Fratesi, G.; Gebauer, R.; Gerstmann, U.; Gougoussis, C.; Kokalj, A.; Lazzeri, M.; Martin-Samos, L.; Marzari, N.; Mauri, F.; Mazzarello, R.; Paolini, S.; Pasquarello, A.; Paulatto, L.; Sbraccia, C.; Scandolo, S.; Sclauzero, G.; Seitsonen, A. P.; Smogunov, A.; Umari, P.; Wentzcovitch, R. M. QUANTUM ESPRESSO: A Modular and Open-Source Software Project for Quantum Simulations of Materials. *J. Phys. Condens. Matter* **2009**, *21* (39). https://doi.org/10.1088/0953-8984/21/39/395502.

(3) Giannozzi, P.; Andreussi, O.; Brumme, T.; Bunau, O.; Nardelli, M. B.; Calandra, M.; Car, R.; Cavazzoni, C.; Ceresoli, D.; Cococcioni, M.; Colonna, N.; Carnimeo, I.; Corso, A. D.; de Gironcoli, S.; Delugas, P.; Jr, R. A. D.; Ferretti, A.; Floris, A.; Fratesi, G.; Fugallo, G.; Gebauer, R.; Gerstmann, U.; Giustino, F.; Gorni, T.; Jia, J.; Kawamura, M.; Ko, H.-Y.; Kokalj, A.; Küçükbenli, E.; Lazzeri, M.; Marsili, M.; Marzari, N.; Mauri, F.; Nguyen, N. L.; Nguyen, H.-V.; Otero-de-la-Roza, A.; Paulatto, L.; Poncé, S.; Rocca, D.; Sabatini, R.; Santra, B.; Schlipf, M.; Seitsonen, A. P.; Smogunov, A.; Timrov, I.; Thonhauser, T.; Umari, P.; Vast, N.; Wu, X.; Baroni, S. Advanced Capabilities for Materials Modelling with Q Uantum ESPRESSO. *J. Phys. Condens. Matter* **2017**, *29* (46), 465901.

(4) Giannozzi, P.; Baseggio, O.; Bonfà, P.; Brunato, D.; Car, R.; Carnimeo, I.; Cavazzoni, C.; De Gironcoli, S.; Delugas, P.; Ferrari Ruffino, F.; Ferretti, A.; Marzari, N.; Timrov, I.; Urru, A.; Baroni, S. Quantum ESPRESSO toward the Exascale. *J. Chem. Phys.* **2020**, *152* (15). https://doi.org/10.1063/5.0005082.

(5) Perdew, J. P.; Burke, K.; Ernzerhof, M. Generalized Gradient Approximation Made Simple. *Phys Rev Lett* **1996**, *77* (18), 3865–3868. https://doi.org/10.1103/PhysRevLett.77.3865.

(6) Hamann, D. R. Optimized Norm-Conserving Vanderbilt Pseudopotentials. *Phys. Rev. B - Condens. Matter Mater. Phys.* **2013**, *88* (8), 085117. https://doi.org/10.1103/PHYSREVB.88.085117/FIGURES/6/MEDIUM.

(7) Troullier, N.; Martins, J. L. Efficient Pseudopotentials for Plane-Wave Calculations. *Phys. Rev. B* **1991**, *43* (3). https://doi.org/10.1103/PhysRevB.43.1993.

(8) Tan, Y. W.; Zhang, Y.; Bolotin, K.; Zhao, Y.; Adam, S.; Hwang, E. H.; Das Sarma, S.; Stormer, H. L.; Kim, P. Measurement of Scattering Rate and Minimum Conductivity in Graphene. *Phys. Rev. Lett.* **2007**, *99* (24). https://doi.org/10.1103/PHYSREVLETT.99.246803.

(9) Langreth, D. C.; Lundqvist, B. I.; Chakarova-Käck, S. D.; Cooper, V. R.; Dion, M.; Hyldgaard, P.; Kelkkanen, A.; Kleis, J.; Kong, L.; Li, S.; Moses, P. G.; Murray, E.; Puzder, A.; Rydberg, H.; Schröder, E.; Thonhauser, T. A Density Functional for Sparse Matter. *J. Phys. Condens. Matter* **2009**. https://doi.org/10.1088/0953-8984/21/8/084203.

(10) Berland, K.; Cooper, V. R.; Lee, K.; Schröder, E.; Thonhauser, T.; Hyldgaard, P.; Lundqvist, B. I. Van Der Waals Forces in Density Functional Theory: A Review of the vdW-DF Method. *Rep. Prog. Phys.* **2015**. https://doi.org/10.1088/0034-4885/78/6/066501.

(11) Thonhauser, T.; Zuluaga, S.; Arter, C. A.; Berland, K.; Schröder, E.; Hyldgaard, P. Spin Signature of Nonlocal Correlation Binding in Metal-Organic Frameworks. *Phys. Rev. Lett.* **2015**. https://doi.org/10.1103/PhysRevLett.115.136402.

(12) Monkhorst, H. J.; Pack, J. D. Special Points for Brillouin-Zone Integrations. *Phys. Rev. B* **1976**. https://doi.org/10.1103/PhysRevB.13.5188.

(13) Eyring, H. The Activated Complex in Chemical Reactions. *J. Chem. Phys.* **1935**, *3* (2), 107. https://doi.org/10.1063/1.1749604.

(14) V. Lucarini; J.J. Saarinen; K.-E. Peiponen; E.M. Vartiainen. *Kramers–Kronig Relations in Optical Materials Research*. http://www.springer.de/phys/books/opticalscience/.

(15) Marinopoulos, A. G.; Reining, L.; Rubio, A.; Olevano, V. Ab Initio Study of the Optical Absorption and Wave-Vector-Dependent Dielectric Response of Graphite. https://doi.org/10.1103/PhysRevB.69.245419.
